# 

**Published:** 1871-06

**Authors:** 


### CONTENTS.

### ORIGINAL COMMUNICATIONS.

PAGE

Hypodermic Medication. By W. Greene, M. D., Americus, Ga . . . . . 129

A Case of Premature Delivery, Induced by Dysentery, without the usual Pain of Labor. By C. D. Smith, of Newnan, Ga . . . . . 146

Communication from J. J. Caldwell, M. D., Zebulon, Ga . . . 148

Extract from the Minutes of the Atlanta Academy of Medicine . . . . . 151

### SELECTIONS.

Detroit Academy of Medicine . . . . . 154

Death from Chloral . . . . . 162

Overdose of Chloral Hydrate . . . . . 163

Observations on Palpitations of the Heart and its Treatment. By Frederick B. Nunnely, M. D., London . . . . . 164

Dangerous and Fatal Results from the use of Hydrate of Chloral. By H. W. Fuller, M. D., F. R. C. P . . . . . 171

Cases of Successful Version, with Living Children, After Failure of the Forceps. By J. J. Phillips, M. D., London . . 174

Castor Oil Soap . . . . . 176

### EDITORIAL.

Medical Clinique of Atlanta Medical College . . . . . 177

Atlanta Medical College—Failure of its Enemies . . . . . 180

On the Wasting Diseases of Infants and Children . . . . . 183

Dr. C. C. Crawford's Pamphlet, and other Kindred Matter . . 184

Proceedings of the Macon Convention . . . . . 186

Alumni Meeting . . . . . 189

In Memoriam . . . . . 190

